# Towards a Mechanistic Model of Tau-Mediated Pathology in Tauopathies: What Can We Learn from Cell-Based In Vitro Assays?

**DOI:** 10.3390/ijms231911527

**Published:** 2022-09-29

**Authors:** Julia Sala-Jarque, Karolina Zimkowska, Jesús Ávila, Isidro Ferrer, José Antonio del Río

**Affiliations:** 1 Molecular and Cellular Neurobiotechnology Group, Institute for Bioengineering of Catalonia (IBEC), Parc Científic de Barcelona, 08028 Barcelona, Spain; 2 Department of Cell Biology, Physiology and Immunology, Faculty of Biology, University of Barcelona, 08028 Barcelona, Spain; 3 Ciberned (Network Centre of Biomedical Research of Neurodegenerative Diseases), Institute of Health Carlos III, 08028 Barcelona, Spain; 4 Institute of Neuroscience, University of Barcelona, 08035 Barcelona, Spain; 5 Centro de Biología Molecular “Severo Ochoa” (CBMSO) CSIC/UAM, 28049 Madrid, Spain; 6 Department of Pathology and Experimental Therapeutics, Facultat de Medicina i Ciències de la Salut, University of Barcelona, 08908 Hospitalet de Llobregat, Spain

**Keywords:** tauopathies, neurodegeneration, seeding, spreading

## Abstract

Tauopathies are a group of neurodegenerative diseases characterized by the hyperphosphorylation and deposition of tau proteins in the brain. In Alzheimer’s disease, and other related tauopathies, the pattern of tau deposition follows a stereotypical progression between anatomically connected brain regions. Increasing evidence suggests that tau behaves in a “prion-like” manner, and that seeding and spreading of pathological tau drive progressive neurodegeneration. Although several advances have been made in recent years, the exact cellular and molecular mechanisms involved remain largely unknown. Since there are no effective therapies for any tauopathy, there is a growing need for reliable experimental models that would provide us with better knowledge and understanding of their etiology and identify novel molecular targets. In this review, we will summarize the development of cellular models for modeling tau pathology. We will discuss their different applications and contributions to our current understanding of the “prion-like” nature of pathological tau.

## 1. Introduction

Tauopathies are a group of over 25 different neurodegenerative diseases (NDs), including, among others, Alzheimer’s disease (AD), primary age-related tauopathy (PART), progressive supranuclear palsy (PSP), chronic traumatic encephalopathy (CTE), Pick’s disease (PiD), corticobasal degeneration (CBD), or globular glial tauopathy (GGT) [1,2,3]. They are all characterized by the progressive accumulation of the microtubule (MT)-associated protein tau (MAPT) into insoluble aggregates. In parallel to tau aggregation, patients suffer from neuron loss and increased cognitive decline. Although considerable efforts have been made to overcome the pathophysiological aspects of tauopathies, there are currently no effective therapies for affected individuals. 

Tau is an MT-binding protein that plays a fundamental role in modulating neuronal MTs dynamics in the axons of neurons [4]. Moreover, it is also present in oligodendrocytes and astrocytes, although with lower expression levels [5,6]. Importantly, under pathological conditions, tau dissociates from MTs and aggregates into highly insoluble, abnormally phosphorylated forms [7,8]. Indeed, tau aggregates are the main histopathological hallmark shared by all tauopathies. However, the primary cell types affected (neurons or glial cells), the regional distribution of the aggregates, and the morphology of these aggregates vary by disease [9] (e.g., storm-like, diffuse plaques, etc.). In AD, for example, tau aggregates form paired helical filaments (PHFs) and straight filaments, which contribute to the formation of neurofibrillary tangles (NFTs) [10]. In addition, tau aggregates from different tauopathies are biochemically distinct, as they differ in their extent of protease resistance, resulting in unique Western blot banding patterns [11,12], or different structures revealed in recent studies using cryo-electron microscopy (cryo-EM) [13]. 

To date, the relationship between tau aggregates and pathogenicity remains unclear. Thus, tau aggregates could either be a co-occurrence of another unidentified underlying disease or a direct cause of neurodegeneration. The latter is supported by the fact that mutations in the *MAPT* gene on chromosome 17 [14] cause inherited frontotemporal dementia with parkinsonism linked to chromosome 17 (FTDP-17) [12,15], implying a causal link between tau malfunction and neurodegeneration. In the case of sporadic human tauopathies, the gradual appearance of tau aggregates positively correlates with neurodegeneration [16]. Moreover, the presence of insoluble tau aggregates during the course of the disease is not limited to specific brain areas but appears to spread throughout the brain. For example, in patients with AD, histopathological tau lesions progress in a predictable, stereotyped, and hierarchical pattern along neuroanatomically connected brain regions (which has been described in neuropathological studies [17,18] and more recently confirmed via positron emission tomography (PET) [19]). However, the specific molecular and cellular mechanisms underlying the progression of tau pathology are still unclear. Since 2009 [20,21], there has been increasing evidence pointing to the belief that tau can spread between cells in a “prion-like” manner. The “prion-like” hypothesis states that seeded aggregation and cell-to-cell spread of pathological tau are two fundamental pathological aspects of human tauopathies (see [22] as a recent example). 

In recent years, experimental approaches based on cellular models (which recapitulate some pathological aspects of human tauopathies) have supported the idea that tau behaves as a “prion-like” protein. Cellular models enable precise and detailed studies of the putative mechanisms responsible for the progression of tauopathies. Understanding such mechanisms is critical not only for getting a better knowledge of the etiology of human tauopathies but also for identifying molecular targets for the development of novel therapies. In this review, we provide a brief overview of the most relevant literature on cellular model systems developed to investigate the prion paradigm of tau pathology. We will discuss their contributions to our current knowledge and highlight some major limitations.

## 2. Tau Structure and Processing

In the healthy adult human brain, there are six tau isoforms ranging from 352 to 441 amino acids in length. These isoforms are the products of alternative mRNA splicing of exons 2, 3, and 10 [23,24]. Exon 10 inclusion results in the production of tau isoforms with four repeats (4R), whereas its exclusion produces isoforms with three repeats (3R). The adult human brain expresses both 3R and 4R tau at equivalent ratios [25]. While tau aggregates in the brains of AD patients maintain this proportion, other tauopathies favor one isoform over the other. Thus, human tauopathies can be classified as 3R, 4R, or 3R/4R (e.g., PiD, GGT, and AD, respectively), determined by the isoforms present in the aggregates [26].

**Figure 1:**
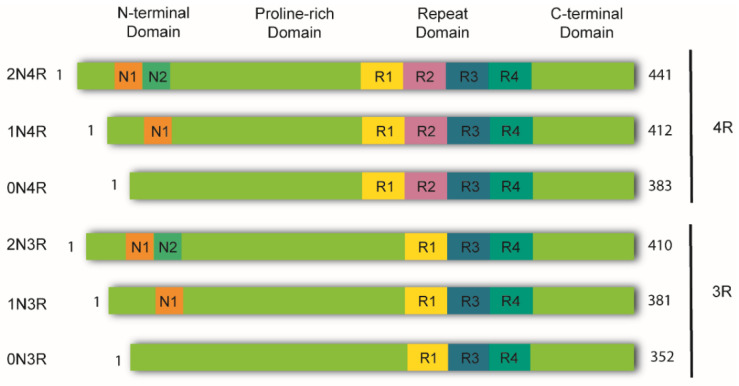
Schematic representation of tau isoforms. In the adult human brain, tau is found as six major isoforms (352-441 amino acids) resulting from alternative mRNA splicing. The N-terminal domain consists of either 0, 1, or 2 inserts encoded by exons 2 and 3 (0N, 1N, or 2N). The proline-rich domain is followed by the repeat domain (RD) also known as the microtubule binding domain (MTBR). Here, inclusion of exon 10 produces tau isoforms with four repeats (4R), whereas its exclusion produces isoforms with three repeats (3R). The RD is followed by the C-terminal domain.

Tau is a highly soluble and intrinsically disordered protein that lacks a well-defined secondary structure [27]. Therefore, depending on interactions with different binding partners, it can adopt various conformations [25]. Despite this, tau can be divided into four functional domains: (a) the N-terminal domain, also known as the projection domain; (b) the proline-rich mid-domain, with different phosphorylation residues; (c) the MT-binding domain (MTBD) or repeat domain (RD), which consists of three (R1, R3, and R4) or four (R1-R4) MTs-binding repeats, due to the absence or presence of exon 10, respectively, and (d) the C-terminal region (Figure 1).

The physiological functions of tau are predominantly regulated by several post-translational modifications (PTMs) [28,29]. Phosphorylation and dephosphorylation regulate the affinity of tau for MTs under physiological conditions [30]. Despite this, hyperphosphorylated tau aggregates are a well-known histopathological marker [31]. Nevertheless, it remains to be validated whether abnormal phosphorylation is indeed the cause of tau aggregation, as there are conflicting reports on this topic (see [32,33] for review).

## 3. The “Prion-like” Nature of Tau and Its Strains

As previously mentioned, the progressive accumulation of tau results in dysfunction in brain regions affected by tau pathology [34]. In the early 1990s, Braak and Braak conducted a cross-sectional neuropathological study of the brains of AD-deceased patients [17] and established that the development of tau pathology occurs in an hierarchical fashion. Their work showed that the progression of NFTs does not occur randomly in the brain. Instead, tau lesions first accumulate in the locus coeruleus, from where they tend to spread to the transentorhinal region of the temporal lobe, followed by the allocortex and neocortex (first in associational areas and later in the primary sensory cortex and the primary motor cortex) [35]. Interestingly, some cellular types appear to be more vulnerable to tau aggregation than other cells [34,36,37,38]. Taken together, these findings could be interpreted in the context of pathological tau spreading in the brain through trans-cellular propagation. 

The idea that tau spreads through cell-to-cell transmission is commonly referred to as the “prion-like” hypothesis. Since 2009, there has been an increasing body of evidence supporting the notion that tauopathies and PrDs share some common pathological mechanisms [3,20,21,39]. Indeed, not only tauopathies but also other NDs with amyloid deposition—also called “neural proteinopathies” (e.g., synucleinopathies)—are now referred to as “prion-like” diseases because they all seem to have replication and propagation processes akin to those observed in PrDs. According to the “protein-only” hypothesis, PrDs are a group of NDs caused exclusively by an infectious protein or prion known as the proteinase K-resistant prion protein (PrPSc) [39], which is the pathological form of the cellular prion protein (PrPC) [40]. Importantly, PrPSc forms insoluble amyloid aggregates that spread across brain regions by cell-to-cell transmission. Nonetheless, “prion-like” diseases are not currently considered bona fide prions [41,42,43] because, unlike prions, there is no conclusive evidence of interindividual transmissibility (see [44]). Therefore, such terminology has been used to specify these proteins that have similar replication and propagation processes as PrDs but are not infectious. Nevertheless, the fact that PrDs and “prion-like” diseases may share some pathological mechanisms could have significant implications for the development of novel treatments.

The “prion-like” hypothesis proposes that, initially, only a small number of neurons initiate the process of tau aggregation. In these cells, tau misfolds and recruits its endogenous counterparts, templating the misfolding state via a process similar to the growth of crystals [45], known as nucleation-dependent polymerization. Replication of the misfolding state is a defining feature of “prion-like” behavior referred to as “seeding,” and the term “seed” describes the minimal unit needed to template the misfolding state of tau. Once the seed is formed, it begins the process of self-seeded fibrillization, in which tau monomers are progressively recruited and added to the growing fibril. Mature tau aggregates (or fibrils) exhibit amyloid properties (e.g., cross-β structure, Thioflavin-positive staining, resistance to detergents). Large tau aggregates can then be fragmented, creating new fibrils or seeds, thereby amplifying the pathology. At this point, competent seeds can be released and propagated to adjacent or synaptically connected healthy cells [33]. The stable propagation of tau aggregates is another characteristic of the prion paradigm behavior and is termed “spreading.”

**Figure 2:**
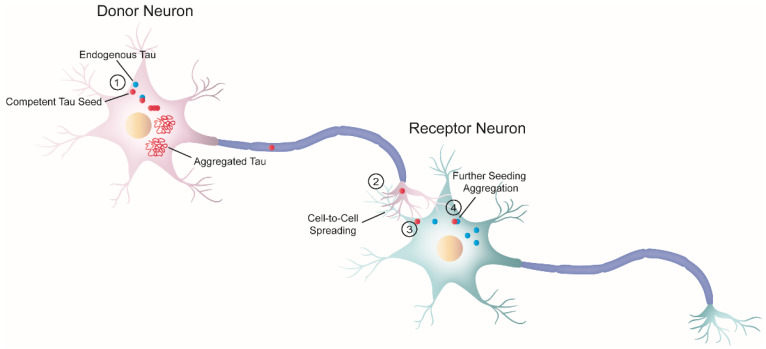
Schematic diagram of cell-to-cell progression of tau pathology. j The formation of tau aggregates begins in a donor neuron (pink) when a misfolded seed-competent tau (red) templates its misfolded state to its endogenous monomeric counterpart (blue), through a process known as seeding. Ultimately, the seeding process produces tau aggregates with amyloid properties. In parallel, tau seeds travel along the axon to the synaptic terminal of the donor neuron. k Once there, tau is released or transferred from the donor neuron to the receptor neuron (greenish-blue). Although not depicted here, glial cells could also internalize misfolded tau seeds. l Next, the receptor neuron internalizes seeded-competent tau. This diagram depicts only one of the several proposed mechanisms related to trans-cellular spreading, in which free tau seeds are released from the axon terminal and are internalized by the receptor neuron through direct membrane fusion. However, numerous studies have proposed a variety of cellular pathways involved in the progression of pathological tau, as reviewed by in steps k and l [46,47,48]. The exact nature of the pathological tau involved in the cell-to-cell transfer process is also unknown, and different groups have proposed a variety of candidates [49,50,51]. m Inside the receptor neuron, pathogenic tau can recruit endogenous cellular tau and seed further tau aggregation. Overall, this process ensures the progression of the pathology.

Both seeding and trans-cellular spreading of proteopathic tau seeds have been proposed as the pathological mechanism to explain the stereotyped progression of neurodegenerative tauopathies. Therefore, since tau is an intracellular protein, cell-to-cell spreading implies, in a broad sense, a four-step process (Figure 2): (1) one cell harboring intracellular tau aggregates (termed donor neuron), (2) release of competent tau seeds from the donor cell into the extracellular space, (3) internalization by a neighboring healthy cell (termed receptor neuron or glia), and (4) once inside, recruitment of their endogenous counterparts initiating further seeding.

It is generally accepted that prion “strains” are the underlying cause of the heterogeneity observed among PrDs. Prion strains are amyloids with different morphologies, known as “conformational variants” or “polymorphs.” They can be classified based on defined incubation times in affected organisms, unique brain lesion profiles of aggregate distribution, and horizontally stable dissemination. Consistent with the “prion-like” hypothesis, current experimental evidence strongly indicates the existence of tau strains [52]. For instance, as previously stated, human tauopathies differ in the tau isoforms present in their inclusions (3R, 4R, 3R/4R [53]) and are characterized by unique trypsin digestion patterns. In addition, inoculating sarkosyl-insoluble tau fractions from different sources (transgenic murine models or human tauopathies) into rodent models triggers the formation of tau aggregates. Such aggregates sometimes fully recapitulate some pathological features of their human counterparts [54]; however, this is not always the case [55]. More recently, cryo-EM studies have revealed that each tauopathy has aggregates with unique disease-specific structures [56,57]. Taken together, it seems plausible that cell-specific amyloidogenic processes produce different tau strains, which may account for the heterogeneity of human tauopathies (e.g., see [52,58] for reviews).

## 4. Principal Approaches in the Rodent Model’s Scenery for Tau Pathology

Researchers have engineered both in vitro and in vivo models, which recapitulate several pathological features of human tauopathies, to study the “prion-like” properties of tau. Although in vivo models are out of the scope of the present review, we have decided to include a brief description of some of the most relevant approaches to illustrate the current trends and motivations in the “prion-like” research field. Detailed information on in vivo approaches can be found in recent reviews [59,60,61].

To generate tau pathology in rodent models, the gold standard has been to develop transgenic mice expressing mutated variants of the human tau protein [61]. For instance, mice expressing mutant human P301L or P301S tau (both FTLD-17 related) under different neural promoters (e.g., *PRNP* or *Thy-1*) robustly develop tau aggregates in their brains over time [62,63]. Consequently, these models have been instrumental in dissecting specific pathological mechanisms involved in tauopathies [64,65]. However, in these animals, virtually all brain cells (or at least all neurons) overexpress, often with several copies, mutated variants of the human *MAPT* gene. Therefore, it becomes almost impossible to differentiate between cell-autonomous and non-autonomous mechanisms of tau propagation [66]. Consequently, various groups have explored alternative strategies to generate alternative models, including mice lacking the expression of endogenous murine tau while overexpressing non-mutated human tau [67,68,69] or restricting the expression of tau-mutated variants in specific brain areas [70,71]. Other approaches involve injecting either viral particles, to mediate the expression of mutated tau with viral vectors [72,73], or seed-competent tau species from various sources (e.g., brain extracts from patients or synthetic recombinant tau fibrils, also known as preformed tau fibrils (PFFs)), as they are able to induce seeding and spreading of endogenous tau even in wild-type mice [3,21,55,69]. 

Despite the improvements, several inconveniences related to in vivo experimentation still exist. Generally, working with animals is time-consuming and quite expensive to maintain, not to mention the putative ethical implications. Reference [74] has put forward the development of reliable, robust, and appropriate cellular models to study human tauopathies. 

## 5. Cellular Models of Tau Pathology: Aggregation, Seeding, and Spreading

For the last 20 years, various cellular models have been developed to study tau aggregation, seeding, and spreading. Such models range from basic cellular organisms [75,76], immortalized mammalian cell lines [20,77], primary neuronal cell cultures [78,79], and, more recently, induced pluripotent stem cells (iPSCs), or organoids [80,81,82]. The reductionist perspective provided by in vitro approaches is subject to certain limitations. Although it makes it easier to isolate complex cellular mechanisms that otherwise would be impossible to address, cellular models are far from recreating the complexity of the brain, which implies the loss of several layers of information. However, they have some advantages over animal experimentation. For instance, cell cultures (a) easier to maintain; (b) have lower intrinsic variability, and (c) can be genetically manipulated, allowing them to overexpress or silence genes of interest faster and easier. Therefore, in vitro models have become an invaluable source of information on our current knowledge of the similarities between tau pathology and PrDs.

### 5.1. In Vitro Modelling of Tau Aggregation: Seminal Models

In the early 2000s, before the “prion-like” hypothesis acquired momentum [20,21], scientists were focused on creating cellular models that provided information related to the process of intracellular tau aggregation (e.g., kinetics, cytotoxicity, tau phosphorylation, tau truncation, etc.) [83,84,85,86]. These seminal works did not involve the presence of an extracellular competent seed to trigger intracellular tau aggregation (Table 1). Therefore, they are not directly related to the study of the “prion-like” paradigm. However, they paved the way for more sophisticated models. Indeed, developing in vitro approaches to study tau pathology has proven not to be an easy task. For instance, wild-type full-length tau is a highly soluble protein that does not readily aggregate in cultured cells under physiological conditions [10,87]. Early studies revealed that the overexpression of wild-type, mutated, or truncated tau variants had a greater propensity to form aggregates within living cells [83]. However, the overexpression of wild-type full-length tau can be hazardous to certain types of cell lines [88]. Because tau binds to MTs, high amounts of soluble tau over-stabilize tubulin, which inhibits cell division and results in cell death [89,90]. To overcome this limitation, certain groups opted to regulate tau expression under doxycycline (Dox)-inducible systems [83,91]. Indeed, a prominent example is that of E. Mandelkow’s group, as they were one of the first to publish the development of a Dox-inducible cell line to regulate the expression of mutated forms of tau [83]. Here, the authors were interested in addressing the cytotoxicity associated with intracellular tau aggregation [83]. Khlistunova et al. genetically engineered the N2a cell line to overexpress one of the following variants: (a) wild-type tau RD; (b) tau RD harboring the ΔK280 mutation (known to facilitate tau aggregation), and (c) tau RD with the ΔK280 mutation, also carrying two additional proline mutations (known to inhibit tau aggregation). They found that tau RD ΔK280 overexpression had a great tendency to be misfolded and to aggregate, which in turn was toxic to the cells [83]. In contrast, the other tau RD variants exhibited little to no aggregation, and their expression was not harmful. This study also showed that either suppressing tau expression or adding specific tau-aggregation inhibitors resulted in reduced cellular toxicity and intracellular tau aggregates. Thus, Khlistunova et al. revealed the utility of cellular models to operate as platforms to screen for tau-directed drugs. Indeed, in 2017 [92], the N2a cell line with the ΔK280 mutation [83] was adapted to screen for 1649 compounds, which led to the identification of novel inhibitors of tau aggregation.

Around the same time, J. Kuret’s laboratory developed an alternative approach in which they generated a HEK293-derived cell line that stably expressed wild-type full-length human tau (Tau40), also under the control of a Dox-inducible promoter [84]. In this study, the authors triggered intracellular tau aggregation by adding Congo red (CR) (an agonist of tau aggregation) to the medium. After seven days of CR treatment, Bandyopadhyay et al. observed a significant increase in the levels of aggregated tau, followed by decreased cell viability. Overall, both studies revealed a link between cell death and tau aggregation, but more importantly, demonstrated that living cells can aggregate tau under specific conditions. 

A significant breakthrough in this field was the introduction of fluorescently tagged tau variants to monitor tau aggregates in living cells. In 2007, the group led by G.V.W. Johnson was the first to include genetically encoded fluorescent probes in cellular models of tau pathology [85,93]. They adapted the technology of Fröster Resonance Energy Transfer (FRET) to directly observe tau–tau intermolecular interactions [85]. Briefly, FRET is a non-radiative energy transfer method in which a donor fluorophore in its excited state interacts with an acceptor fluorophore in the ground state when the distance between them is less than 10 nm. The authors engineered the HEK293 cell line to express either Tau40 or truncated tau (Tau1-421). Each construct was tagged with cyan fluorescent protein (CFP) or yellow fluorescent protein (YFP) at either the N- or C-terminal of the tau protein. Chun and Johnson triggered intracellular aggregation by inducing tau phosphorylation and then quantified the FRET signal to evaluate tau association levels [85]. Together, the data demonstrate the applicability of FRET technology to visualize and quantify tau interactions. Since then, many others have successfully introduced this technique to their cellular models [95,96,97,98,99]. 

Similarly, in a second study from the same group, the authors addressed tau–tau interactions through an alternative method called the “bimolecular fluorescence complementation” (BiFC) assay [93]. The BiFC assay involves a fluorescent protein split into two non-fluorescent fragments. When the two fragments are close enough, they form a protein complex that emits a fluorescent signal. Chun et al. engineered HEK293 cells to co-express the non-fluorescent green fluorescent protein (GFP) fragment (GFP1-10), and Tau40 fused to a complementary but non-fluorescent GFP fragment (GFP11). In their study, under basal conditions, monomeric soluble GFP11 fragment was accessible to the GFP1-10 [93]. Therefore, both fragments could assemble, consequently exhibiting high fluorescence intensity levels. By contrast, the induction of tau aggregation decreased the GFP signal due to the steric hindrance of the two molecules. For this reason, this BiFC assay is referred to as the “turn-off” approach. Indeed, because, under basal conditions, cells emit the highest GFP intensity, early aggregation events cannot be detected. To overcome this limitation, Tak et al. proposed the BiFC “turn-on” assay [94]. The authors developed a Venus-based BiFC technique by having the Tau40 fused to the N- and C-terminal fragments of the Venus fluorescent protein (VN173 and VC155, respectively). In this case, soluble tau exhibited little fluorescence, whereas aggregated tau produced a significant increase in the BiFC signal, thereby allowing detection of early events of tau aggregation. After that, other groups introduced the “turn-on” approach to their experimental designs [100,101]. 

Taken together, these two works adapted cutting-edge optical techniques to monitor tau interactions in real-time and opened new perspectives to the field. Moreover, they inspired other groups to also include fluorescently tagged tau variants in their cellular models. Ultimately, this led to significant improvements in the technology used to visualize tau, along with the realization of the potential applications of these novel cellular approaches.

### 5.2. Cellular Models of Tau Seeding: Cellular Internalization of Proteopathic Tau Seeds

In the late 2000s, multiple experimental approaches were developed simultaneously, and several groups engineered mammalian cell lines to express different variants of the tau protein. However, it was unclear whether extracellular pathogenic tau could be internalized into healthy cells and trigger intracellular tau aggregation through the seeding process. To test this, researchers treated their cellular models with extracellular seed-competent tau derived from various sources, such as preformed fibrils (PFFs) made of synthetic recombinant tau, human brains, and mouse brains. In this section, we will review the most relevant models developed to scrutinize the seeding phenomenon in the context of living cells (Table 2).

The 2009 paper published by M.I. Diamond’s lab established that cultured cells could internalize extracellular tau PFFs, inducing endogenous tau aggregation and subsequent transmission of the aggregates between co-cultured cells [20]. For this experiment, Frost et al. engineered two cell lines, C17.2 and HEK293, to transiently express either tau RD or Tau40, each fused to YFP. Importantly, the expression of either construct was not enough to trigger aggregation within the cells, as they remained soluble in the cytoplasm. In contrast, the addition of extracellular fibrillated tau (but not monomeric tau) to the medium induced the fibrillization of the endogenous Tau40-YFP, thereby increasing the levels of intracellular fluorescence. Furthermore, in 2009, Clavaguera et al. [21] provided similar evidence in vivo. Briefly, Clavaguera and colleagues injected pathogenic tau derived from the brain of a transgenic mouse model of human tauopathy into the brain of an AD mouse model that does not develop tau aggregates. The inoculation of pathological tau triggered not only the formation of tau aggregates, but also the spread along synaptically connected brain regions. Collectively, both studies proved for the first time that tau could seed aggregation and propagate trans-cellularly, providing strong support for the “prion-like” hypothesis. Consequently, scientists began to comprehend the potential of the “prion-like” paradigm, and other important contributions soon followed.

For example, in 2010, Nonaka et al. engineered SH-SY5Y cells to transiently express either tau 1N3R (3R) or 1N4R (4R) [102]. To increase the levels of intracellular tau aggregation, the authors successfully evaded the endocytic process by delivering 3R, or 4R-tau PFFs with Lipofectamine^TM^. In this study, tau seeding only occurred when the extracellular and endogenous tau shared the same isoform. Nonaka et al. argued that an asymmetric seeding barrier between 3R and 4R tau conditioned the seeded polymerization. In support of this, in 2016, S.B. Prusiner’s group reported similar results [103], this time using fluorescently tagged tau constructs. Woerman et al. modified the HEK293 cell line to express tau RD of either four (4RD) or three repeats (3RD), harboring the mutations P301L and V337M or L226V, respectively, and fused to YFP. The two cell lines were named Tau(4RD*LM)-YFP(1) [3] and Tau(3RD*VM)-YFP [103]. Eventually, the group created two additional cell lines, the Tau(3RD*VM,4RD*LM)-YFP, which was engineered to express both plasmids [103]; and the Tau(4RD*LM)-YFP(2), which the Tau(4RD*LM)-YFP(1) modified to produce higher levels of tau 4RD. To induce intracellular tau aggregation, the authors incubated each cell line with brain extracts from 4R, 3R, or 4R/3R human tauopathies. Woerman and colleagues reported that 4R tauopathies could only trigger aggregation in the Tau(4RD*LM)-YFP(1) cell line. Similarly, 3R tauopathies only induced tau aggregation in Tau(3RD*VM)-YFP cells. Surprisingly, 3R/4R tauopathies (CTE and AD) were unable to seed aggregation in neither cell line, but triggered tau aggregation in Tau(3RD*VM,4RD*LM)-YFP cells and Tau(4RD*LM)-YFP(2). Ultimately, these data support the idea that an asymmetric barrier exists between tau isoforms, which can be considered additional proof of the existence of tau strains. 

Another interesting strategy was soon published by J.L. Guo and V. Lee, who also provided relevant data pointing to the still-new idea that exogenous tau aggregates could recruit their soluble endogenous counterparts [77]. Their cellular model consisted of the QBI-293 cell line transiently transfected with either Tau40 or Tau40, harboring the mutation P301L (T40PL). To induce intracellular aggregation, Guo and Lee added tau PFFs to the cellular medium, along with a protein delivery system named “BioPORTER reagent” to increase the number of internalized tau seeds. As a result, both cell lines produced tau aggregates with a variety of morphologies, a phenomenon that, until then, had never been reported in a cellular model [77]. Guo and Lee interpreted this as different stages of tau aggregation. However, we speculate that these differently shaped aggregates were tau strains formed simultaneously within the cells. Additionally, the authors reported an asymmetric seeding barrier between P301L and wild-type tau, analogous to previously published studies described above [102,103]. 

In a follow-up study, Guo and Lee updated the above-mentioned cellular model [77] by adding a tetracycline-regulated promoter to control T40PL expression and a GFP flag (T40PL-GFP) [91]. Thus, the system was optimized for real-time monitoring of tau aggregates. The authors exogenously supplied seed-competent tau to trigger intracellular fibrillization. Remarkably, live-cell imaging of GFP-tagged tau inclusions revealed that tau aggregates are “dynamic” entities undergoing “fusion” and “fission” events, rather than “static” structures. Thus, this study further elucidated the molecular properties of newly formed tau aggregates within living entities. In addition, Guo and coworkers found that the cells propagated tau aggregates throughout their lineage over several generations without causing any obvious cytotoxicity, which is in striking contrast to previous reports [83,84]. Nevertheless, it is plausible that their discrepancies could be the result of different experimental designs. However, despite limitations, more recently, the T40PL-GFP cell line [91] proved to be a useful tool for performing high-throughput screenings of potential anti-tau treatments [104]. Indeed, cellular models with fluorescently tagged tau have been shown to be suitable for screening for tau inclusion inhibitors, as will be discussed below. 

As a last example, Mirbaha et al. wanted to address the identity of the minimal tau assembly that upon internalization could trigger intracellular tau aggregation [49]. To test this, the authors adapted the “split-luciferase complementation assay” (SLC) to report on the presence of tau aggregates, using an experimental design analogous to the one described by Tak et al. [94]. Briefly, their SLC approach was based on the subunits CLuc and NLuc, which upon reconstitution, regain their luciferase activity and, in the presence of D-luciferin, produce the luminescence signal. Mirbaha et al. engineered the HEK293 cell line and neural primary cell cultures to express the tau RD fragment harboring the P301S mutation, either fused to CLuc or NLuc (RD(P310S)-NLuc/CLuc). Interestingly, Mirbaha and colleagues used tau assemblies purified from AD brains and identified tau trimers (n ≥ 3) as the minimal seed to induce tau aggregation in both HEK293 and primary neuron cultures. Remarkably, larger fibrils also produced an increased luciferase signal, which was not observed after the addition of monomeric tau species, suggesting that monomeric tau does not have “prion-like” properties. Since then, other groups have also attempted to identify the nature of such minimal tau assembly, failing to deliver consistent results, as will be discussed below. Finally, regarding the use of SLC assays in the study of tau pathology, there are different examples available in the literature [105,106]. Notably, SLC approaches are less adopted than fluorescent strategies, as the formers do not allow for direct optical visualization of tau aggregates, although most of them display a considerable high dynamic range compared to fluorescent assays.

Altogether, these studies confirmed that extracellular seed-competent tau could be internalized by cells and seed the formation of intracellular tau aggregates, thus providing experimental evidence for the “prion-like” hypothesis. Furthermore, the authors also reported, for the first time, fundamental pathogenic, and mechanistic insights related to the seeding process. For example, the asymmetric seeding barrier between tau isoforms or the ability to pass aggregates from mother to daughter cells. However, the results from these experiments can be difficult to compare and interpret, as the authors used different: (a) cell lines, (b) tau mutations, (c) methods to detect/quantify intracellular tau aggregates, and d) seed-competent tau species to trigger aggregation. It is worth mentioning that here the term “seed” includes a variety of tau species that may not be comparable in terms of their relevance to the pathophysiological process of human tauopathies. Ideally, intracellular aggregation should be triggered by human-derived seed-competent tau. However, researchers opted for using PFFs due to the limited availability of human samples. As previously mentioned, PFFs are composed of recombinant synthetic tau monomers that are often truncated isoforms with aggregation-prone mutations. In this regard, PFFs are usually assembled in cell-free environments in the presence of polyanionic factors (i.e., heparin and RNA) for tau efficient aggregation [107,108]. Although the reaction of fibrilization produces bona fide tau amyloid fibrils, the cell-free environment lacks multiple cellular mechanisms (i.e., chaperones and kinases) that are present in the cellular milieu. Therefore, it is not surprising that increasing evidence indicates that PFFs are dramatically different from patient-derived fibrils, and thus their translational value has been questioned [109]. Therefore, it would be crucial to examine whether the results obtained using PFFs are replicated when more disease-relevant tau seeds are used. 

Despite these limitations, the cellular models presented here have numerous advantages and have proven to be a useful strategy for investigating not only the pathological mechanisms underlying tau self-seeded fibrilization, but also their applicability in compound screening. 

#### Cell-Based Assays: Proteopathic Seeding

In parallel to the development of cellular models to study cellular mechanisms related to the seeding process, researchers also generated cell-based assays to examine other aspects of the seeding properties of tau. Cell-based assays can be defined as in vitro tools that produce a detectable signal within a living cell. In the study of human tauopathies, cell-based assays have been engineered to detect the presence of seed-competent tau in samples from different sources (e.g., human brains). Indeed, cell-based assays have been instrumental in understanding the relationship between seeding and pathology, also known as proteopathic seeding [96].

The first cell-based assay, specifically designed to detect and quantify tau proteopathic seeding, was produced in M.I. Diamond’s lab in 2014 [96], as a reporter cell line based on FRET technology (FRET biosensor cell line). The authors genetically engineered HEK293 cells to stably overexpress the tau RD fragment bearing the P301S mutation, fused to either CFP or YFP. Importantly, both constructs remained soluble in the cytoplasm with background fluorescence levels under basal conditions. By contrast, the addition of extracellular seed-competent tau (e.g., brains of patients with AD) triggered the intracellular aggregation of the RD-CFP/YFP constructs, thereby intensifying the FRET signal. In addition, delivering tau aggregates with Lipofectamine to the cells increased the sensitivity of the assay by 100-fold, as the authors were able to detect tau aggregates down to femtomolar concentrations through flow cytometry. In summary, Holmes et al. created a reliable, sensitive, and sensible tool to detect proteopathic tau seeding. The second generation of the FRET biosensor cell line was recently released by M.I. Diamond’s group [98]. Upgrades included: (a) replacement of the previous human ubiquitin C promoter with a human cytomegalovirus promoter, and (b) exchange of CFP and YFP for mCerulean3 (Cer) and mClover3 (Clo) sequences (tauRD(P301S)-Clo/Cer) [98]. Hitt et al. isolated two monoclonal cell lines, which, based on the intensity of the produced signal, were named version 2 low (v2L) and version 2 high (v2H). Remarkably, the second generation of biosensors is more sensitive than the first, as they detect proteopathic seeding at much lower concentrations [98]. 

Finally, the FRET biosensor cell lines can be adapted for a wide range of experimental applications. For instance, M.I. Diamond’s lab used the first generation of FRET biosensors to demonstrate that tau proteopathic seeding correlates with the pathology progression and can be detected before the appearance of classical histopathological markers in both P301S mice and patients with AD [92,104,105]. These findings strongly suggest that tau seeding is an important diagnostic biomarker for human tauopathies. Hence, there would clearly be value in introducing the FRET biosensor cell line as an assay for routine diagnostic purposes. However, one of the biggest challenges is to detect proteopathic seeding in samples collected from living patients. Indeed, previous studies have reported inconclusive and contradictory results regarding this issue [98,110]. A second example is developing cell models of tau pathology to identify tau-based treatments, as already mentioned, leading to the identification of several small molecular compounds that inhibit the formation of tau inclusions [92,104]. In this line, several groups have exploited the FRET biosensor cell line for drug discovery. For example, previous work by Seidler et al. proved the suitability of the cell-based biosensor assay to test the performance of various peptides as inhibitors of proteopathic tau seeding [111]. Although the contributions of the FRET biosensor cell line are undeniable, there is one major limitation that we believe should be mentioned. The existence of asymmetric seeding barriers has been reviewed in the present manuscript [102,103]. Because the FRET biosensor cell line overexpresses only one tau construct (P301S RD-CFP/YFP), it could be possible that the detection of seed-competent tau species was biased towards certain tau conformers due to asymmetric seeding barriers. Thus, it would be of great value to address the presence of seed-competent tau species in different reporter cell-based assays expressing different tau variants, to diminish the probability of reporting false negatives. In summary, there is increasing interest in producing and improving similar cellular models for providing platforms to develop diagnostic and therapeutic strategies [104,112].

### 5.3. Cellular Models of Tau Spreading and Serial Propagation

Tau is an intracellular protein; therefore, the “prion-like” paradigm needs to explain how pathological tau can be transferred between cells to determine the cellular mechanisms involved in the process. As previously described, the very first report on trans-cellular propagation of tau aggregates was published by Frost et al. [20]. In subsequent work, Kfoury et al. provided direct evidence of serial propagation of tau aggregation between co-cultured cells [95]. Here, the authors engineered the HEK293 cell line to produce two distinct cellular populations. The donor population expressed tau RD bearing the P301L/V337M mutation fused to CFP, and the receptor population expressed an identical construct but tagged to YFP. To probe serial propagation, Kfoury et al. triggered intracellular tau aggregation into the donor population before co-culturing both cell lines. After 48 h, the authors detected an increased FRET signal in the receptor cells, which reported the presence of CFP/YFP tau aggregates. These results indicate that tau seeding had occurred in the receptor population through direct protein contact [95]. Importantly, Kfoury et al. showed that cell-to-cell transfer of tau aggregates could be blocked by the addition of anti-tau antibodies [95]. In a separate study, the same group reported that the internalization occurred through the binding to heparan sulfate proteoglycans [113]. Collectively, these findings provide strong evidence that tau spreading may occur extracellularly in different ways, providing a theoretical framework to explain the pattern of progression observed in AD patients. 

In this section, we have seen examples that illustrate how cell lines have helped to prove that tau can be propagated trans-cellularly. However, cell lines lack the complexity that characterizes neural cells. For instance, the idea that the progression of tau pathology occurs trans-synaptically implies the possibility that particular proteins involved in tau spreading are exclusively expressed by neural cells. Therefore, studying whether and by which mechanisms tau spreads from one cell to another should be addressed in more relevant models, such as primary rodent neurons. 

### 5.4. Tauopathies in Primary Neural Cells: The Use of Microfluidic Devices in Experimental Design

**Figure 3:**
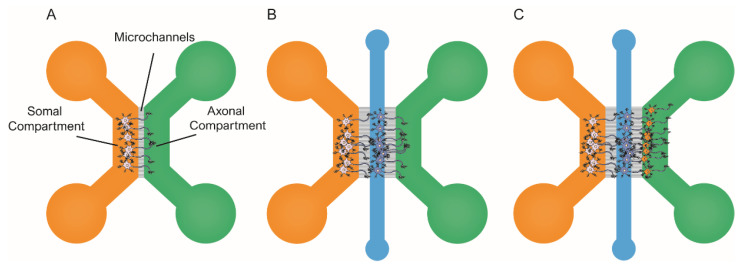
Schematic representation of the most commonly used microfluidic platforms for modeling tau spreading. All these models allow for the isolation of soma/axons, along with treating each channel independently. (**A**) schematic representation of the two-chambered model [79] shows the architecture of the microfluidic device. Neural cells are seeded in the soma compartment (orange). After several, only the axons have been able to grow and reach the axonal compartment (green); (**B**) schematic representation of the three-chambered model that allows for the co-culture of two populations of primary neurons [119]. Here, the third chamber (blue) is used to seed the second population; (**C**) schematic representation of the three-chambered model to co-culture three independent neural populations [78].

Neurons are post-mitotic cells with highly specialized morphology, which makes them greatly different from mammalian cell lines. Therefore, not only cellular pathways, but also the expression of specific proteins may differ from those in cell lines. Regarding tauopathies, this is illustrated by the fact that tau is mainly expressed by neurons (see above), as their function is to regulate MT dynamics. Thus, under physiological conditions, tau is localized in the axons. By contrast, under pathological conditions, it can be detected in the perikaryon and distal dendrites. Since most cell lines do not recreate neuronal morphology, these pathological events cannot be reproduced, thereby questioning the relevance of some published findings. Nevertheless, acknowledging such limitations has contributed to developing experimental approaches based on rodent neural primary cell cultures [114]. For instance, researchers have developed viral vectors to easily infect primary neurons to induce the overexpression of wild-type or mutated forms of tau. In addition, the introduction of microfluidic devices to experimental designs has helped characterize tau cell-to-cell spreading in primary neurons (e.g., see [115]). Briefly, in this context, the most commonly used microfluidic platforms include two or more chambers fluidically isolated but interconnected by microchannels [116,117], as illustrated in Figure 3. This design allows fluidic isolation of the neuronal soma and dendrites from axons. Therefore, researchers can independently treat, tune, and monitor the different chambers. Additionally, it is possible to co-culture neural populations in isolation within the distinct compartments and recreate neural connections, which can help investigate tau spreading between synaptically connected neurons [118]. 

Some groups have focused on understanding which tau species are involved in the cell-to-cell spreading of tau pathology. One of the early accounts to address this issue in primary neurons was the 2013 published study by Wu et al. [79]. They used microfluidic devices to prove that both axonal and the somatodendritical compartments were able to endocytose tau PFFs (Figure 3A). In addition, Wu et al. provided evidence of tau transport along the axons, both anterogradely and retrogradely [21,91,116]. The authors claimed that neurons could only internalize low-molecular-weight (LMW) tau aggregates, but not monomeric tau or larger fibrils. Conceptually similar work was pursued by B. Hyman’s group in 2015 [119]. In this case, they used a three-chambered microfluidic device, in which the two first compartments were seeded with primary neurons (Figure 3B). In the study, Takeda et al. identified a rare soluble high-molecular-weight (HMW) phosphorylated tau (isolated from AD) brains as the most efficiently internalized species and the key to tau propagation. Indeed, various studies have reported different tau species as the culprits of tau spreading [20,49]. However, the use of different cellular types, the distinct origin of the tau seeds used to induce aggregation, and the unequal tau isolation protocols adapted in these experiments could explain the lack of consistency in the identified species. Moreover, it is possible that there is not a unique seed-competent species, and it may even be different among tauopathies and individual patients [120].

Other groups have focused on the cellular mechanisms involved in cell-to-cell spreading, as the pathological events by which competent tau seeds are released into the extracellular space are still unknown. For example, Calafate et al. used microfluidic devices to co-culture a monoclonal HEK293 cell line that harbored intracellular tau aggregates (referred to as donor cells) as they stably expressed TauP301L-GFP, along with primary hippocampal neurons (referred to as receptor cells), either expressing: TauP301L or HA-TauP301L [78]. Of note, the experimental design is conceptually similar to that previously published by Kfoury et al. [95]. The donor cell line released competent tau seeds, resulting in the formation of tau aggregates within the neural population. Next, the authors engineered the donor cell line to express the synaptoproteins Ngl1 and LRRTM4, as they can form synaptic-like structures in non-neuronal cells [121,122]. This resulted in increased synaptic-like contacts between the two cell populations. Calafate et al. reported a significant rise in tau propagation, suggesting a link between synapse and tau spreading. To confirm the role of synapses in the interneuron transfer of tau, the authors used a microfluidic device model with three independent chambers (Figure 3C). Researchers seeded each compartment with primary neurons that grew axons to the adjacent chambers, thereby forming synaptic connections. Tau aggregation was induced only in the first population (donor cells), while the other two remained unaltered (receptor cells). During the next two weeks, receptor populations in the second and third chambers accumulated pathogenic tau, consistent with the cell-to-cell transfer of tau aggregates. Overall, these findings indicate that synapses contribute to the spreading of tau pathology. 

Wu et al. conducted a similar study in which they used three-chambered microfluidic devices with three neural populations to assess tau spreading [118] (Figure 3C). The authors seeded primary neurons expressing tau P301S RD fragment fused to YFP within the microfluidic chambers. Researchers induced tau aggregation in the first neural population (referred to as donor cells), whereas the other two remained unaltered (referred to as receptor cells). Thanks to the fluorescently tagged tau, the authors could track the spreading of pathological tau throughout the three synaptically connected neural populations. The researchers claimed that pre-synaptic neurons did release tau and transferred it to the recipient cells via the extracellular medium (previously suggested by [78,95]). Finally, Wu et al. demonstrated that increased neural activity promoted tau secretion, proving that neural activity enhances tau spreading. Overall, these studies [78,118] and others [113] strongly suggest that the progression of tau pathology involves extracellular tau seeds as the principal species involved in cell-to-cell propagation. Indeed, there are multiple accounts of studies where tau spreading in transgenic AD rodent models was successfully blocked upon the addition of anti-tau antibodies. 

### 5.5. iPSCs for Modeling and Studying Tauopathies In Vitro

**Figure 4:**
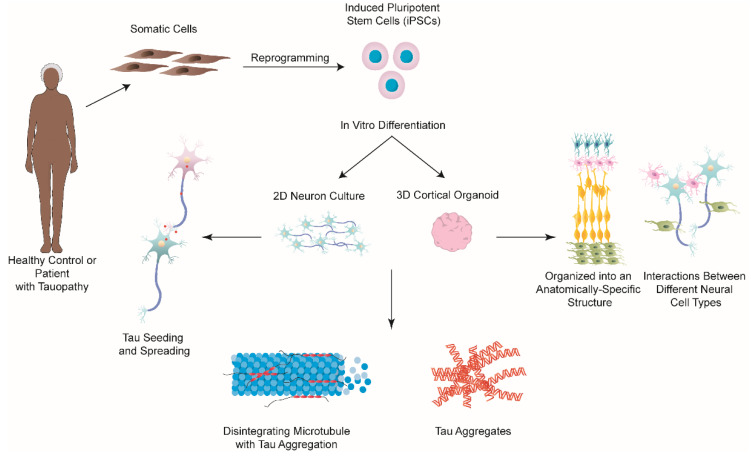
iPSCs-based 2D and 3D approaches for modelling tauopathies in vitro. Somatic cells, such as fibroblasts, can be taken from patients with either a tauopathy or healthy controls, and be reprogrammed to iPSCs, which can subsequently be differentiated into various types of neurons. Both 2D and 3D approaches have been studied to model different types of tauopathies. Studies utilizing these two models have successfully shown the formation of tau aggregates along with neurodegeneration. 2D neuron cultures have also been used to examine the mechanisms behind tau seeding and spreading. However, 2D cultures lack the intricate microenvironment and structural arrangement of the human brain. 3D brain organoids are comprised of a variety of different neuronal cell types, including neurons, neural progenitors, oligodendrocytes, and astrocytes, which organize into an anatomically-specific structure that closely mimics that of the developing human brain. This model allows for examining the interactions between different neuronal cell types, which better models the processes that occur in vivo compared to 2D models. The continued improvement of these iPSC-based technologies will contribute to a better understanding of the pathological mechanisms involved in tauopathies and will hopefully lead to the development and discovery of effective treatments against them.

Until recently, the majority of research focused on understanding tauopathies has utilized transgenic mouse models, immortalized cell lines, as well as primary rodent neurons. However, although these studies have provided us with invaluable insight into understanding tauopathies, these models cannot fully recapitulate most aspects of human disease due to genetic heterogeneity. In this regard, iPSCs and derived technologies have revolutionized in vitro models of human diseases, including NDs [123,124]. The great advantage of iPSCs in neurodegenerative research is that they can be induced from any cell type and be differentiated into different types of neurons, as well as glia [125,126,127,128,129,130]. Furthermore, patient-derived iPSCs maintain the genetic information of donors and have been shown to replicate the disease phenotype in vitro, without external manipulation [131,132]. Therefore, in the last decade, researchers have turned to iPSCs to model and study tauopathies (Figure 4), with many studies focusing on AD.

Most of the studies published to date on this topic have focused on trying to utilize iPSCs to see if they can simply model tau pathology in iPSC-derived neurons (Table 3). For example, Israel and colleagues were one of the first to show that iPSC-derived neurons obtained from reprogrammed fibroblasts from AD patients with both the sporadic and familial form of the disease, were able to replicate some of the disease hallmarks including significantly higher levels of phospho-tau (Thr231), as well as Aβ(1/40) and active glycogen synthase kinase-3β (aGSK-3β) [131]. Other studies, rather than obtaining cells from patients, have created wild-type iPSCs carrying various mutations that would allow the study of different phenotypes of tau pathology [133,134,135]. Verheyen et al. developed a genetic model of FTDP-17 by introducing two *MAPT* mutations, pathogenic IVS10+16 mutation and pro-aggregant P301S point mutation in exon 10, using zinc-finger nucleases (ZFNs) [135]. The mutated iPSC-derived neurons expressed 4R tau and, although they did not observe spontaneous tau aggregation, they were able to induce tau oligomerization following tau 4RD (P301L) seeding [135]. Furthermore, the study observed increased apoptosis, increased frequency of Ca^2+^ bursts, as well as increased lysosomal pH in the neurons carrying the P301S mutation [135]. Therefore, these studies demonstrated that iPSCs can be used to effectively model and observe the relevant phenotypes of tauopathies in vitro, although the majority of these diseases take decades to manifest in patients.

Following the development of adequate iPSC-based tauopathy models, researchers began to focus on examining the seeding, spreading, and aggregation of tau in these models (Table 3). In 2015, Usenovic et al. were one of the first to explore if tau spreading occurs in healthy human iPSC-derived neurons following seeding with different tau species and the effects that would have on these neurons [82]. They seeded the neurons with two types of tau seeds: full-length monomers and oligomers, which were obtained from full-length recombinant, wild-type human tau [82]. Through a set of elegant experiments, Usenovic and colleagues observed an increase in phosphorylated pathological tau and intracellular tau aggregates following tau oligomer, but not monomer, treatment [82]. Furthermore, they observed that tau aggregates shared many characteristics with NFTs from AD patients’ brains and they were shown to cause synapse loss, neurite retraction, an imbalance in neurotransmitter release, as well as abnormal Ca^2+^ homeostasis in the iPSC-derived neurons [82]. Altogether, the results from this study supported previous work, conducted on animals and cell lines, that tau oligomers are toxic species that propagate tau pathology and neurodegeneration [139,140,141]. In a different study, Oakley et al. developed an in vitro longitudinal single-cell live-imaging system to visualize the effects of tau seeds on tau aggregation and neurotoxicity within single, patient-derived iPSC neurons [81]. In order to visualize this, the iPSC neurons were transduced with a lentivirus that stably expressed a non-spontaneously aggregating TauRD fused to a YFP biosensor. Then, tau seeds extracted from mice with a MAPT P301L mutation were seeded with the cells to observe if they caused aggregation of the TauRD construct. Using this system, the researchers were able to compare neurotoxicity between iPSC-derived neurons which developed tau aggregates and those that did not in the same well. Through this approach, the downstream toxicity of tau uptake and aggregation was able to be analyzed, while also being able to control for the TauRD construct expression as well as for the exposure of the iPSC neurons to seed-competent tau. This technology allowed the researchers to observe that the neuronal uptake and propagation of tau seeds significantly reduced neuron cell viability [81]. Additionally, they found that tau aggregation occurred more rapidly in iPSC neurons from AD patients with the PSEN1 L435F mutation [81]. This innovative method, together with the ability to obtain iPSCs with the patient’s genetic background, opens up the possibility to investigate differences in tau-induced neurotoxicity in different tauopathies. Lastly, an extensive study led by Manos et al. optimized the development of wild-type human iPSC-derived cortical neurons, which do not require tau overexpression in order to form insoluble tau aggregates [136]. They found that these differentiated neurons, when seeded with sarkosyl-insoluble AD brain seeds, had a significantly increased amount of endogenous tau aggregates after 7 weeks, which was observed to be concentration-dependent [136]. They also observed that sarkosyl-insoluble seeds were more potent at causing tau aggregates than crude AD lysate seeds [136]. Interestingly, in contrast to sarkosyl-insoluble material from AD brains, sarkosyl-insoluble material from healthy control brains had no effect on tau aggregation in the iPSC-derived cortical neurons, indicating that templating is specific to AD material [136]. Through the combination of human cortical neurons with the ability to endogenously express tau with competent tau species derived from AD patient brains, this study has created a physiologically relevant tau seeding model for studying tau pathology, which would not be able to be achieved with animal models nor cell lines, thus showcasing how powerful of a research tool iPSC technology is. Additionally, it is worth mentioning that iPSC models can also allow us to study the nonlinear relationship between pathological tau and Aβ production [142], which could help us to better understand their interactions in AD. 

All of the aforementioned studies utilized 2D culture models and, although they have revealed several of the hallmark features of tauopathies, they lack the intricate microenvironment and structural arrangement of the human brain. Therefore, to create a more physiologically and structurally relevant in vitro model of the human brain and, thus, a tauopathy model, recent research has focused on creating 3D-based cell culture models including brain organoids. In comparison to 2D models, 3D models of the brain allow for interactions between different neural cell types, and they are also capable of mimicking perfusion (and diffusion-based molecular transport) something that is impossible to model in 2D culture [143]. Moreover, a brain organoid development process closely mimics that of the developing human brain as it organizes into an anatomically-specific structure [144]. This could be of benefit when studying tauopathies, such as AD in which tau has been observed to spread in a highly stereotyped pattern across the brain [17,18], to investigate the spreading of tau in particular brain regions that could be modeled by organoids. Thus, many studies have used patient-derived iPSC to create brain organoids that replicate AD pathology, mainly Aβ aggregates and hyperphosphorylated tau (Figure 4) (Table 3) [76,142,143]. For example, Gonzalez and colleagues created cerebral organoids from iPSCs derived from patients with early-onset AD and observed that these organoids spontaneously developed structures comparable to Aβ plaques and NFTs after 110 days in vitro [80]. However, to date, there have been no studies examining tau seeding and spreading in brain organoids. Similarly, the studies published to date have only focused on whether or not tau aggregates can form in these organoids and, if β- and γ-secretase inhibitors can reduce tau pathology [137,138], but not on specific molecular and cellular mechanisms underlying the progression of tau pathology, which are still not understood. Therefore, more studies are required on these topics. However, the high heterogeneity between organoids, long culture time (often >100 days), lack of vasculature, and the inability to obtain the full cytoarchitectonics that are observed in the human brain (limited number or lack thereof of oligodendrocytes and microglia) are just some of the limitations that researchers are faced with when working with organoids (see below) [145,146].

## 6. Conclusions

In summary, we have reviewed some of the most significant cellular models developed to study the “prion-like” properties of tau pathology. Although they have some limitations, cellular models have provided a better understanding of the pathological mechanisms of human tauopathies. Engineered based on different strategies, the introduction of fluorescent probes to monitor and visualize tau aggregation in real-time has been instrumental to most of the current applications of such models. Immortalized cell lines have proven to be excellent platforms for drug discovery and may become excellent tools for diagnostic purposes. However, immortalized cell lines may not be suitable to study the process of tau spreading, since it is now believed that synapsis plays a major role in the progression of tau pathology. Therefore, unique experimental paradigms such as the combination of primary neurons and microfluidic devices have provided tremendous insight into the tau species involved in proteopathic seeding along with the role of synapses and neural activity in the progression of tauopathies. More recently, the introduction of iPSCs and organoids has opened new perspectives in the field. Since iPSCs can be derived directly from human patients and maintain specific disease phenotypes, they may become a key translation approach prior to in vivo validation of therapeutic approaches. Indeed, the majority of recent literature studying tau pathology is focusing on brain organoids—created using iPSCs—and, having compared the different existing models, the authors cannot argue that this approach is well worth focusing on as it most closely resembles human physiology, instead of being a ‘human-like’ or ‘similar’ model. However, despite of the many advantages of human brain organoid studies described above and in Table 4, several developments of this culture method are still required in order to improve the system for the study of tau-mediated brain pathology (see [147]). Nevertheless, some recent studies have been capable of both generating vascularized brain organoids [148] and incorporating myelinating oligodendrocytes in human cortical spheroids [149], thus bringing the model even closer to fully recapitulating the human brain. Collectively, all of the aforementioned in vitro models, together with the continued development of new technologies, will hopefully lead to uncovering more detail about the cellular mechanisms involved in tauopathies and thus contribute to the development of better therapeutics for patients. 

## Figures and Tables

**Table 1:** Seminal works in cellular models of tau aggregation.

Tau Isoform	Variants	Host Cell	Expression	Aggregation Inducer	Detection Method	Reference
4RD	Wild-type ΔK280 ΔK280/PP (I277P/I308P)	N2a	Stable doxycycline-inducible	Spontaneous formation	ThS ICC	[83]
2N4R	Wild-type	HEK293	Stable doxycycline-inducible	Congo red	ICC	[84]
0N4R Tau1-421	Wild-type	HEK293	Transient expression	Spontaneous formation Phosphorylation	FRET (CFP/YFP)	[85]
0N4R 4RD	ΔK280 I277P/I308P	HEK293	Transient expression	Spontaneous formation Phosphorylation	BiFC (Split GFP)	[93]
2N4R	Wild-Type	HEK293	Stable expression	Phosphorylation	BiFC (Split Venus)	[94]

Abbreviations: CFP: cyan fluorescent protein; BiFC: bimolecular complementation; FRET: Fröster resonance energy transfer; GFP: green fluorescent protein; ICC: immunocytochemistry; ThS: Thioflavin S; YFP: yellow fluorescent protein.

**Table 2:** Relevant cellular models of tau seeding.

Tau Isoform	Variants	Host Cell	Expression	Extracellular Tau	Detection Method	Reference
4RD Tau40	Wild-type	HEK293 C17.2	Transient expression	Tau PFFs	YFP ICC	[20]
1N3R 1N4R	Wild-type	SH-SY5Y	Transient expression	Tau PFFs	GFP ICC	[102]
3RD 4RD	L226V/V337M P301L/V337M	HEK293	Transient expression	Crude brain homogenates from human AD, PiD, CTE, AGD, CBD, and PSP patients	YFP	[103]
Tau40	Wild-type ΔK280 P301L R406W	QBI-293	Transient expression	Tau PFFs	ICC	[77]
Tau40	P301L	QBI-293	Stable doxycycline-inducible	Tau PFFs	GFP ICC	[91,104]
4RD	P301S	HEK293	Stable expression	-Tau PFFs -Tau assemblies purified from AD patients	SLC (NLuc/CLuc)	[49]
4RD	P301S	HEK293	Stable expression	-Tau PFFs -AD brain lysate -P301S brain lysate	FRET (CFP/YFP)	[96]
4RD	P301S	HEK293	Stable expression	-Tau PFFs -AD brain lysate -AD CSF	FRET (Clo/Cler)	[98]
4RD	ΔK280 P301L/V337M ΔK280/I22P /I308P	HEK293	Transient expression	Tau PFFs	FRET (CFP/YFP) Antibody	[95]

Abbreviations: AD: Alzheimer’s disease; AGD: argyrophilic grain disease; CBD: corticobasal degeneration, Cer: mCerulean3; CFP: cyan fluorescent protein; Clo: mClover3; CSF: cerebrospinal fluid; CTE: chronic traumatic encephalopathy; FRET: Fröster resonance energy transfer; GFP: green fluorescent protein; ICC: immunocytochemistry; PFF: preformed fibrils; PiD: Pick’s disease; PSP: progressive supranuclear palsy; SLC: split-luciferase complementation; YFP: yellow fluorescent protein.

**Table 3:** Relevant iPSC and 3D organoid models used to study tau pathology.

Model	Cell Source	Type of Tau Seed	Detection Method	Reference
2D	Neurons derived from wild-type hiPSCs with two *MAPT* mutations	K18 fibrils (P301L)	AlphaLISA	[135]
2D	Wild-type hiPSC- neurons	Full-length human tau monomer and oligomer seeds	ICC ThS	[82]
2D	Familial AD patient hiPSC- neurons expressing a tau aggregation biosensor	Tau seeds derived from mice carrying the MAPT P301L mutation (rTg4510)	in vitro longitudinal single-cell live-imaging system	[81]
2D	-*MAPT*-wild-type hiPSC- neurons -*MAPT*-P301S/E10 + 16 hiPSC-neurons	-Sarkosyl-insoluble material from AD brains -Sarkosyl-insoluble material from healthy control brains	ICC HTRF	[136]
3D (cerebral organoid)	-Wild-type hiPSC-neurons -Familial AD patient hiPSC-neurons	Spontaneous formation	ICC	[80]
3D (cells in Matrigel)	ReNcell human neural stem cells with familial AD mutations APPSL and PS1ΔE9	Spontaneous formation	ICC Modified Gallyas silver staining	[137]
3D (cerebral organoids)	-Wild-type hiPSC-neurons -Familial AD patient hiPSC-neurons	Spontaneous formation	ICC ThS	[138]

Abbreviations: AD: Alzheimer’s disease; hiPSC: human induced pluripotent stem cells; HTRF: Homogeneous Time Resolved Fluorescence; ICC: immunocytochemistry MAPT: microtubule-associated protein tau; pTau: phosphorylated tau; ThS: Thioflavin S.

**Table 4:** Major advantages and disadvantages of reviewed tauopathy models.

Model	Advantages	Disadvantages
In vivo: rodent models of tauopathies	Transgenic models reproduce many of the tau pathologies seen in the brains of human patients	Most transgenic models do not entirely mimic the hallmarks of sporadic human tauopathies in terms of the morphology of tau aggregates and the affected cell types
	The overexpression of mutated forms results in a rapid and robust tau pathology	Most transgenic models rely on the overexpression of mutant tau in virtually all brain cells, making tau spreading studies nearly impossible
	Allow for the evaluation of behavioral impairments	Time consuming and expensive
	Inoculation models of patient-derived material are highly translationally relevant models as they allow the investigation of tau spreading	Not suitable for high-throughput approaches
	They include the complexity of the nervous system, improving their translational value compared to other models	Difficult to monitor tau aggregation and spreading with high spatiotemporal resolution
2D mammalian immortalized cell lines	Rapid experimental turnaround time	Most models do not reproduce neuronal phenotypes
	Easy to culture and transfect	Models that partially differentiate to neuronal phenotypes (i.e., SH-SY5Y) are cancer-derived cells
	Labeling techniques are easily introduced to monitor and track aggregate formation with spatiotemporal resolution	Most models are not complex enough to produce transnationally relevant results regarding tau spreading
	Excellent platforms for high-throughput approaches such as drug screening, especially in monoclonal cell lines	They do not reproduce the complexity of the nervous system
Microfluidic devices: murine neural cells	Excellent platforms for the study of tau spreading as they allow to track the movement of tau aggregates across synapses	Laborious to prepare and maintain
	Ideal platform for spatiotemporal separation of neuronal populations, allowing neural network modeling	High levels of variability between independent experiments (e.g., different litters)
	Small reaction volumes needed	Difficult to transfect
2D iPSC-derived neurons	Maintain the genetic information of donors and can replicate the disease phenotype of the donor in vitro	Lack of complexity
	Easily gene-edited to express tau mutations	Neuronal immaturity
	Tau seeds can be easily introduced to the culture	In vitro differentiation induced heterogeneity
	Labeling techniques are easily introduced to monitor and track aggregate formation with spatiotemporal resolution	Labor- and time-intensive generation and characterization
	Excellent platforms for high-throughput approaches such as drug screening	Lack of intercellular communication between different cell types
3D Cerebral organoids	Maintain the genetic information of donors	Highly variable culture protocols, which can lead to varying outcomes between groups
	Can replicate the disease phenotype without genetic manipulation i.e., spontaneous tau phosphorylation/aggregation	Lack of vasculature
	Closely recapitulate the laminar organization of the developing human cortex and thus can model tau spreading in a more physiologically relevant manner	High variability of tau expression between organoids
	Allow for interactions between different neural cell types	Oligodendrocytes and microglia are often not well formed
	Viable for much longer than neural cells in 2D-culture, allowing the study of long-term effects of tau pathology	More difficult to monitor and track aggregate formation with spatiotemporal resolution due to the dense 3D tissue
	Capable of mimicking perfusion and diffusion-based molecular transport	Can develop a necrotic core caused by lack of oxygen and nutrient diffusion into the inner-most layers
	Can be used to study endolysosomal trafficking abnormalities that affect tau pathology	Labor- and time-intensive generation and characterization

## Data Availability

Not applicable.
